# Water Soaking Disorder in Strawberries: Triggers, Factors, and Mechanisms

**DOI:** 10.3389/fpls.2021.694123

**Published:** 2021-07-20

**Authors:** Grecia Hurtado, Moritz Knoche

**Affiliations:** Institute for Horticultural Production Systems, Leibniz University Hannover, Hannover, Germany

**Keywords:** rain damage, water soaking, *Fragaria* × *ananassa* Duch, cracking, leakage, cuticle, microcrack

## Abstract

Water soaking is an important surface disorder of strawberries that limits unprotected field production. The objective was to identify the mechanism(s) of water soaking. Symptomatic fruit show pale, deliquescent patches of skin. This damage extends into the flesh. Numerous cuticular microcracks occurred in water-soaked areas. Water soaking occurred only if the skin was exposed to liquid water. Water soaking was more rapid when the cuticle had been abraded. Water soaking, anthocyanin leakage, and water uptake all increased with incubation time. There was a lag phase for water soaking and anthocyanin leakage, but not for water uptake. Susceptibility to water soaking increased with fruit ripening and mass. Incubation in isotonic PEG 6000 increased cuticular microcracking but decreased water soaking and water uptake. Incubation in hypotonic fruit juice (natural and artificial) increased water soaking incidence and severity but reduced water uptake. Incubation in dilute citric and malic acids increased plasma membrane permeability as indexed by anthocyanin leakage and increased water soaking. Thus, water soaking involves cuticular microcracking, localized water uptake, bursting of cells, and the release of organic acids into the apoplast. The damage propagates from cell to cell.

## Introduction

Strawberry is a soft fleshy fruit of global importance (Hummer and Hancock, 2009). It is highly perishable, but nevertheless grown mainly in the open field where fruit quality is often severely compromised by rain. The main disorders arising from rain are water soaking and cracking (Herrington et al., 2009). With water soaking, the appearance of the fruit surface is compromised, with the skin looking lighter-colored and deliquescent (Herrington et al., 2009). With cracking, easily visible, gaping cracks occur that often extend deep into the flesh. Both disorders may be observed on different regions of the fruit surface, the calyx, neck, shoulder, or tip (Herrington et al., 2011). Both water soaking and cracking increase the incidence of fruit rots pre- and post-harvest. Together, these disorders result in significant economic loss due not only to the lost opportunity (reduced yield) but also to the additional labor involved in harvesting, grading, and packing a fruit population high in individuals suffering compromised quality (Herrington et al., 2009). In addition, the loss at the consumer level increases.

As a result of these limitations, strawberry production is slowly shifting from open field, to semi- or fully protected cultivation in plastic tunnels or greenhouses. This shift is occurring particularly in areas where the incidence of rainfall is high during the later stages of fruit growth and harvest. However, the additional costs of the protection structures and of energy increase the production costs (Khoshnevisan et al., 2013). Furthermore, not all strawberry cultivars are suitable for production in tunnels where, in susceptible cultivars, protected cultivation sometimes increases the incidence of powdery mildew, spider mites, and calcium deficiency disorders (Grijalba et al., 2015).

The mechanistic bases of water soaking and cracking in strawberries have not yet been properly determined. However, such understanding is the obvious prerequisite to the development of effective countermeasures that mitigate water soaking and cracking through the introduction of innovative cultural methods and/or breeding. The objective of this study was to identify the triggers, factors, and mechanisms underlying the water soaking disorder in strawberry fruit.

## Materials and Methods

### Plant Material

Strawberry fruit (*Fragaria* × *ananassa* Duch., cultivars Clery, Faith, Florentina, Malwina) were harvested from commercial plantings at Gleidingen (lat. 52°16′ N, long. 9°50′ E) and Bad Nenndorf (lat. 52°21′ N, long. 9°20′ E). The change in cultivars was necessary, because in strawberry a given cultivar is available at the optimum stage of ripeness only for a limited period of time. Unless otherwise specified, fruits were harvested randomly at commercial ripeness (>80% of the fruit surface red), placed in a foam tray, and selected for uniformity of size, shape, and color and for freedom from visual defects. Care was taken to not touch the fruit surface. The pedicel was cut to a maximum length of 5 mm. Fruits were processed fresh on the day of sampling or held at 2°C and 80% RH for no longer than 1 day. Previous studies showed that holding fruit for up to 2 days under these conditions had no effect on rates of water uptake or transpiration (data not shown). Unless otherwise specified, the calyx was removed from the fruit by carefully pulling the tip of the calyx toward the pedicel. This occasionally required the fruit to be held down by holding on to the pedicel. In most instances, gravity was sufficient for the fruit to remain in the foam tray during the removal of the calyx. The fruit surface was not touched by hand during the entire procedure. The pedicel stump and the attachment zones of the calyx represent openings that are accessible for water uptake. To exclude artifacts, these holes were sealed using a fast-curing, non-phytotoxic silicone rubber (SE 9186 Clear; Dow Corning Corp., Midland, United States). This procedure is illustrated in Supplementary Figure 1.

### General Procedure

The water soaking damage was induced by incubating fruit in deionized water (one fruit per 100 mL) at room temperature. The fruits were forced under water using a soft plastic-foam plug. After a period of immersion, the fruits were carefully blotted using the soft tissue paper. The extent of water soaking was quantified using a 5-point rating scale or by direct measurement using image analysis. The 5-point rating scale was as follows: score 0 = no water soaking; score 1 = <10% of the surface water-soaked; score 2 = 10–35%; score 3 = 35–60%; and score 4 = >60% of the fruit surface area water-soaked.

The water-soaked fruit surface was inspected by light and fluorescence microscopy. Fruit were incubated in 0.1% acridine orange (Carl Roth, Karlsruhe, Germany) for 3 min, then rinsed with deionized water, and carefully blotted. The fluorescent tracer acridine orange penetrates any microscopic cracks in the cuticle, but not an intact cuticle (Peschel and Knoche, 2005; Becker and Knoche, 2012b; Khanal et al., 2021). The fruit surface was then inspected under incident white and incident fluorescent light using a binocular microscope (Leica MZ10F with filter GFP plus 480–440 nm excitation, ≥510 nm emission; Leica Microsystems GmbH, Wetzlar, Germany). Furthermore, surface scans of water-soaked regions and non-water-soaked control regions were prepared at ×500 using a digital microscope (VHX-7000; Keyence, Osaka, Japan) and coaxial illumination.

Water uptake into submerged fruit was quantified gravimetrically. Fruits were incubated individually in deionized water. Before each weighing, a fruit was carefully blotted dry using a soft paper tissue, then weighed (CPA225D; Sartorius, Göttingen, Germany), and thereafter immediately returned to the incubation solution for a further period. Unless specified otherwise, the number of replicates was 15.

### Experiments

The **effect of partial immersion in water on water soaking** was investigated in “Florentina” fruit. Fruits were incubated for 24 h in deionized water such that (a) one half of the fruit was submerged (longitudinal axis horizontal), or (b) just the fruit tip (longitudinal axis vertical), or (c) just the calyx end (longitudinal axis vertical).

The **effect of wounding on water soaking** was investigated in “Florentina” fruit. The treatments were (a) an incision 5 mm long, 3 mm deep using a razor blade; (b) a hole 1.6 mm diameter and 5 mm deep; and (c) abrasion of the cuticle with carborundum powder (grain size 1,200; Schriever, Hamburg, Germany) of about 10 mm^2^. Treated fruits were then incubated in deionized water for 3 h, and calibrated photographs were taken immediately thereafter. The soaked areas around the wounds were quantified by image analysis (cellSens Dimension 1.7.1; Olympus Soft Imaging Solutions, Münster, Germany). The numbers of replicates were 10.

**Different procedures to quantify water soaking** were compared using “Florentina” fruit. These procedures included the comparison of the rating scheme (above) with direct measurement of water-soaked areas using image analysis. In designs involving repeated measures, to exclude artifacts resulting from repeated blotting, the experiment was run twice: First, using independent measurements and ratings (destructive sampling), and second, using repeated observations (measurements and ratings) on the same fruit (non-destructive sampling). For the repeated measurements, the fruit was rotated and photographed from opposite sides at each time interval. Only when the area of water soaking exceeded 60% of the total fruit surface was the fruit peeled and the peel flattened on a glass plate. A calibrated photograph was taken (Canon DS126271; Canon Inc., Tokyo, Japan). The water-soaked area was quantified using image analysis (cellSens Dimension 1.7.1; Olympus Soft Imaging Solutions, Münster, Germany). The minimum number of replicates was 60.

The **time courses** of change in the water-soaked area, in the absorbance of the incubation solution, and in the mass of water uptake were determined over a 10-h incubation period. The water-soaked area was quantified using image analysis (cellSens Dimension 1.7.1; Olympus Soft Imaging Solutions), and the water uptake was measured gravimetrically as described above. The leakage of anthocyanin into the incubation medium was determined by measuring the absorbance of the incubation medium at 520 nm using a spectrophotometer (Specord 210; Analytik Jena, Jena, Germany). Since the absorbance of an anthocyanin solution depends on pH, the pH was first adjusted to pH 2.3 using citric acid at a final concentration of 37 mM.

**Relationships between water soaking, microcracking, and water uptake** were studied by incubating “Clery” fruit for 0, 2, 4, 8, 16, and 24 h in deionized water. Water soaking was quantified using the rating scheme described above. Microcracking of the cuticle was indexed by quantifying the area infiltrated with acridine orange using fluorescence microscopy. The fruits were dipped in 0.1% (w/w) aqueous acridine orange (Carl Roth, Karlsruhe, Germany) for 5 min. Thereafter, fruits were rinsed, blotted dry, and viewed at ×6.3 under a fluorescence binocular microscope (MZ10F; Leica Microsystems, Wetzlar, Germany). Four calibrated images within randomly selected microscope “windows” were taken (Camera DP71; GFP-plus filter, 480–440 nm excitation, ≥510 nm emission wavelength) per fruit on a total of 10 fruit. The area infiltrated by acridine orange was quantified using image analysis (cellSens Dimension 1.7.1; Olympus Soft Imaging Solutions, Münster, Germany). A tissue infiltrated with acridine orange exhibits orange, yellow, and green fluorescence (Peschel and Knoche, 2005). To quantify the infiltrated areas, the appropriate color thresholds were selected, and all images were batch-processed using the same settings for these thresholds. The infiltrated areas were expressed as a percentage of the area of the microscope window. Water uptake was determined gravimetrically.

The **site on the fruit surface** where water soaking first appeared was identified in “Florentina” strawberries. Fruits were immersed in deionized water and continually inspected for symptoms of water soaking. When a fruit exhibited water soaking, it was cut into four slices of equal thickness and perpendicular to its longitudinal axis. The slice in which symptoms first appeared was recorded. Two batches of fruit were inspected: (a) one with the calyx present and (b) one with the calyx removed. The total number of replicates was 92 per treatment. The osmotic potentials of the juice expressed from the different slices from the same batch were measured. A fruit was cut into two halves along its longitudinal axis. One half was used to express the juice to determine the mean osmotic potential of the fruit. The other half was cut transversally into four slices of equal thickness. The osmotic potential of the expressed juices was determined by water vapor pressure osmometry (VAPRO 5600; Wescor, Utah, United States).

A “gaping assay” was carried out to **evaluate the strain relaxation** in the different regions of the fruit surface. A cut 5 mm long and 2 mm deep was made using a razor blade. Calibrated photographs (Lumix DMC-G80; Panasonic Corporation, Osaka, Japan) were taken on a macrostand immediately after the cut had been made and again 24 h later. Gape width was measured by image analysis. Fruits was maintained at 100% RH during the assay to minimize transpiration. The experiments were carried out using “Florentina” fruit. The number of replicates was 10.

The **effect of ripeness on water soaking** was studied in the fruit of “Florentina.” Fruit were selected at six stages of ripeness as indexed by color, ranging from white to dark red (CM-2600 d, orifice 3 mm diameter; Konica Minolta, Tokyo, Japan). All fruit were incubated for 6 h to induce water soaking. Water soaking was quantified using the rating scheme described above. Water uptake was measured gravimetrically (CPA225D; Sartorius, Göttingen, Germany). The osmotic potentials (VAPRO 5600; Wescor, Utah, United States) of the expressed juices were analyzed from the fruit of the same batch.

The **effect of fruit size on water soaking** was investigated by incubating fruit of five different size classes in water for 6 h. The size classes were <15, 15–20, 20–25, 25–30, and 30–40 g. Water soaking was rated as indicated above. Color (CM-2600 d, orifice 3 mm diameter; Konica Minolta, Tokyo, Japan) and soluble solids (°Brix) using a refractometer (DR6200-T; A. Kruess Optronic, Hamburg, Germany) were determined. This experiment was carried out using “Malwina” fruit. The total number of replicates was 92.

The **effect of water uptake on water soaking** was studied by incubating “Clery” strawberries in solutions of polyethylene glycol 6000 (PEG 6000) or in deionized water. The PEG 6000 solution was prepared to be isotonic to the juice expressed from the fruit of the same batch. The time courses of change in water-soaked area and in water uptake were established by sampling fruit at 0, 1, 2, 4, 6, 8, 11, and 24 h. In addition, the fruit surface was inspected for microcracks as described above.

The role of the osmolytes contained in expressed strawberry juice in the phenomenon of water soaking was addressed in two different experiments.

We analyzed the **effect on water soaking of expressed strawberry juice (natural) and of a synthetic juice** prepared by combining pure solutions of the five major strawberry osmolytes (artificial). Natural juice was expressed from the fruit of the same batch as used in the experiment. The osmolytes in the artificial juice and their relative amounts were glucose (30.3%), fructose (33.2%), sucrose (7.6%), citric acid (9.7%), malic acid (5.7%), and potassium applied as KOH (9.6%) (Herrmann, 2001). Natural and artificial juice was used at “full” (isotonic with the fruit) or “half strength” (half isotonic). Osmotic potentials of the solutions were measured by water vapor pressure osmometry (VAPRO 5600; Wescor, Utah, United States). Deionized water served as control. Fruits were incubated for 4 h, and the water-soaked area was then quantified. The rates of water uptake were determined gravimetrically on fruit from the same batch at 0.5-h intervals for up to 1.5 h. The rates of water uptake were calculated (mg h^−1^) on an individual fruit basis, from the slope of a linear regression fitted through a plot of increasing fruit mass vs. time. The experiment was carried out using “Faith” fruit.

In the second experiment, the **effect of the individual major osmolytes** of “Florentina” strawberry, i.e., of glucose (121.0 mM), fructose (132.7 mM), sucrose (30.4 mM), citric acid (38.9 mM), and malic acid (22.6 mM), on water soaking and the rate of water uptake was established. The solution's concentrations were derived from the composition of the isotonic artificial juice of fruit from the same batch. Deionized water and the isotonic artificial juice were used as controls. The water-soaked area and the rate of water uptake were determined as described above.

The **role of malic and citric acid** in water soaking was studied using a leakage assay and anthocyanin as an indicator of cell membrane damage in “Florentina” (Winkler et al., 2015). Cell walls were stressed to varying extents by incubating fruit in solutions of PEG 6000 of different osmotic potentials, and a time course of anthocyanin leakage was established. Cylinders of the outer flesh and fruit skin were excised using a biopsy punch (8 mm diameter), and these were cut to 2 mm length, rejecting the skin, using parallel razor blades. Flesh disks were blotted and rinsed, and then incubated in isotonic PEG 6000 solution, with and without (control) 39 mM citric and 23 mM malic acid. Disks were removed from solution after 1, 2, 4, 8, and 24 h. The absorbance of the incubation medium was quantified at 520 nm using a spectrophotometer (Specord 210; Analytik Jena, Jena, Germany). Before measuring, the pH of the solution used as a control was adjusted by adding the same volume of acids (after incubation was terminated) as that present in the treatment solution. In this way, the control and treatment solutions had the same pH (pH 2.5), and there was no confounding in anthocyanin detection due to variable pH. In a subsequent experiment, disks were incubated in PEG 6000 solutions of osmotic potential 0, −0.6, −1.2, −1.8, or −2.4 MPa with and without the two acids. Disks were removed from the solutions after 4 h, and the anthocyanin content of the incubation medium was measured as described above. Six disks were excised per fruit and used as paired observations. Three disks represent one replicate. The experiment was carried out using 10 replicates.

### Data Analyses

All experiments were conducted and analyzed using completely randomized designs. Data were analyzed by analysis of variance and linear regression. Means were compared using Tukey's studentized range tests (*p* < 0.05) and the statistical software R (version 3.5.1; R Foundation for Statistical Computing, Vienna, Austria). Unless individual observations are shown (e.g., **Figure 3C**-inset, **Figures 4A,B**-insets, and **Figure 4D**), data are presented as means ± standard errors.

## Results

**Figure 1:**
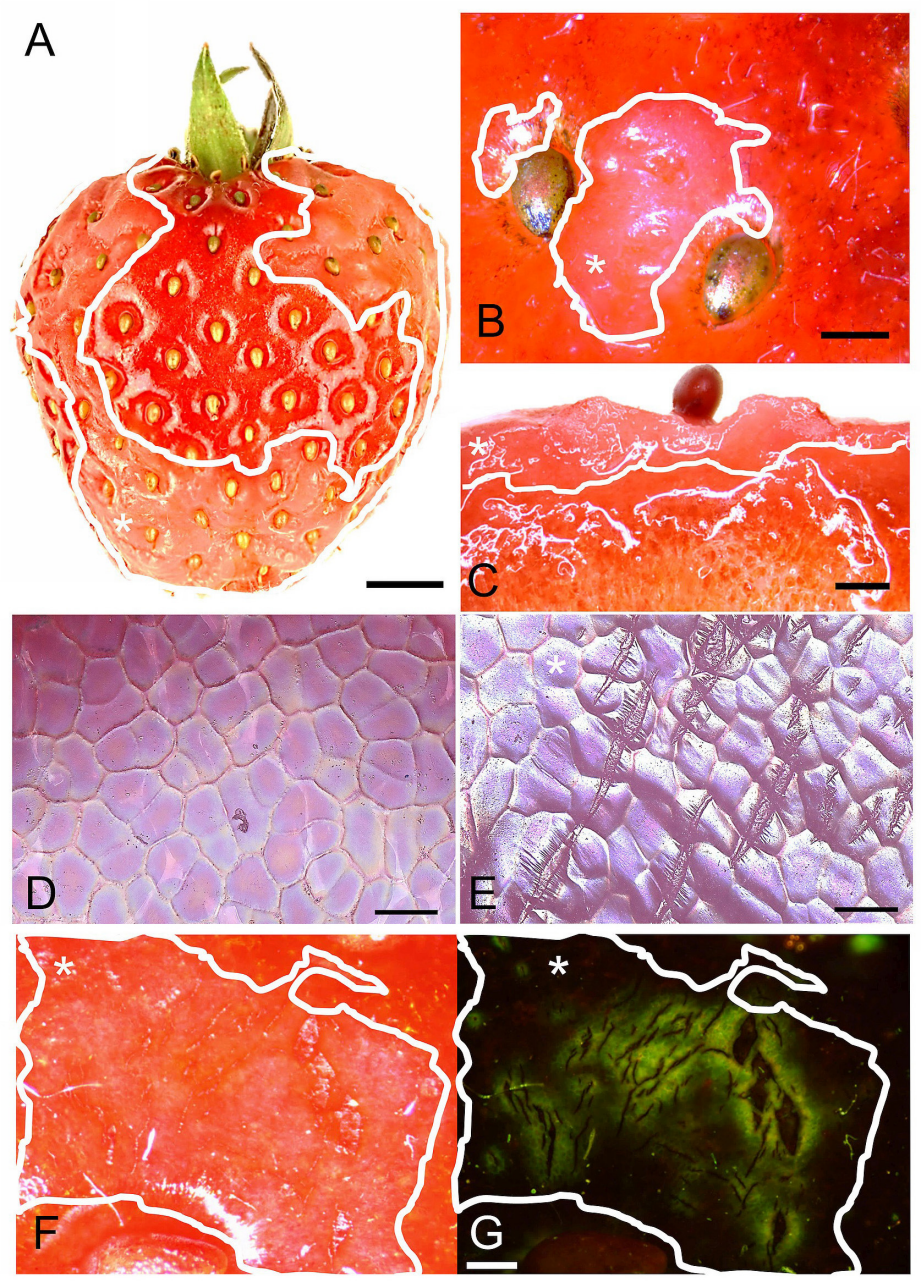
**(A)** Macroscopic view of fruit with water soaking symptoms; **(B)** Detail of achenes with surrounding water-soaked area viewed under a light microscope; **(C)** Micrograph of cross-section of tissue with water soaking. **(D,E)** Scans of the surface in a digital microscope of non-treated and water-soaked fruit with numerous microcracks. **(F,G)** Micrographs of developing symptoms of water soaking viewed under incident **(F)** and fluorescent light **(G)**. Microcracks were infiltrated with acridine orange. The penetrated fluorescent tracer appears as a green fluorescence around a microcrack in the cuticle. Water soaking symptoms are marked by a solid white line. White asterisks in **A**, **B**, **C**, **E**, **F** and **G** identify the water-soaked area. Scale bar in **(A)** = 5 mm, **(B,C)** = 1 mm, **(D,E)** = 0.1 mm and in **(F,G)** = 0.5 mm.

Water soaking in strawberries appeared as irregular patches of skin that are pale, deliquescent, and sometimes pinkish. At times, they looked slightly translucent compared to the shiny, dark-red controls (Figure 1A). The symptoms usually started on the margin between the depressions of adjacent achenes. When severe, the patches covered a major portion of the fruit surface (Figures 1A,B). Water soaking was not limited to the fruit skin but extended several mm below the surface into the flesh (Figure 1C). Scans of the fruit surface and fluorescence microscopy of water-soaked fruit revealed the presence of numerous microcracks in the water-soaked areas (Figures 1E–G). There were no or only few microcracks in non-treated control fruit (no water soaking) (Figure 1D). Water soaking was never associated with fungal development during the short incubation periods of our experiments.

**Figure 2:**
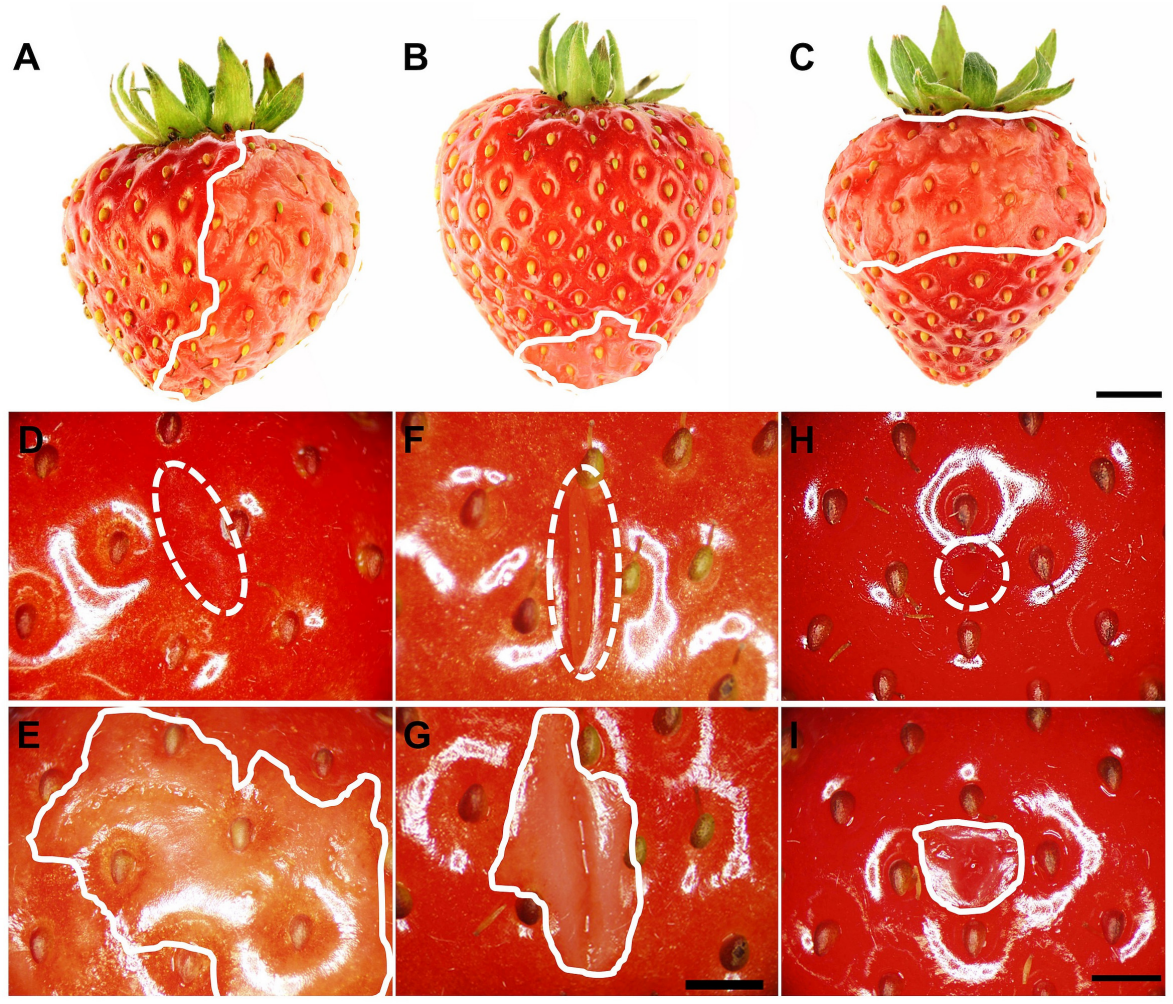
**(A–C)** Induction of local water soaking symptoms by partial immersion of a strawberry fruit in water. **(A)** Half of the fruit (long axis horizontal), **(B)** tip or **(C)** calyx end of the fruit (long axis vertical). Water soaking following abrasion of the cuticle (~10 mm^2^), **(D,E)** incision of the skin using a razor blade (5 mm length ×3 mm depth), **(F,G)** and puncture of the skin using a dissection needle (1.6 mm diameter and 5 mm depth) **(H,I)**. **(D,F,H)** images taken immediately after wounding (*t* = 0 h); **(E,G,I)** images of same area taken after a 3-h incubation in deionized water. The fruit was submerged completely. The water-soaked area around the wound averaged 179.4 ± 48.8 mm^2^ **(E)**, 37.5 ± 5.7 mm^2^ **(G)**, and 20.7 ± 4.2 mm^2^ **(H)**. Scale bar in **(A–C)** = 5 mm and **(D–I)** = 2.5 mm. The dashed white line indicates the wound area, and the solid white line indicates the margin of the water-soaked area. The area outside of the solid white line was also in contact with deionized water and serves as control for a non-water-soaked surface.

Water soaking was always localized and limited to the regions in direct contact with the incubation solution. It never occurred in regions above the water surface when fruit was partially submerged (Figures 2A–C). Water soaking rapidly developed at a wound. Abrading the cuticle using carborundum powder was highly effective in inducing water soaking (Figures 2E–I). Incisions made using a razor blade or puncturing the fruit skin were markedly less effective in inducing water soaking.

**Figure 3:**
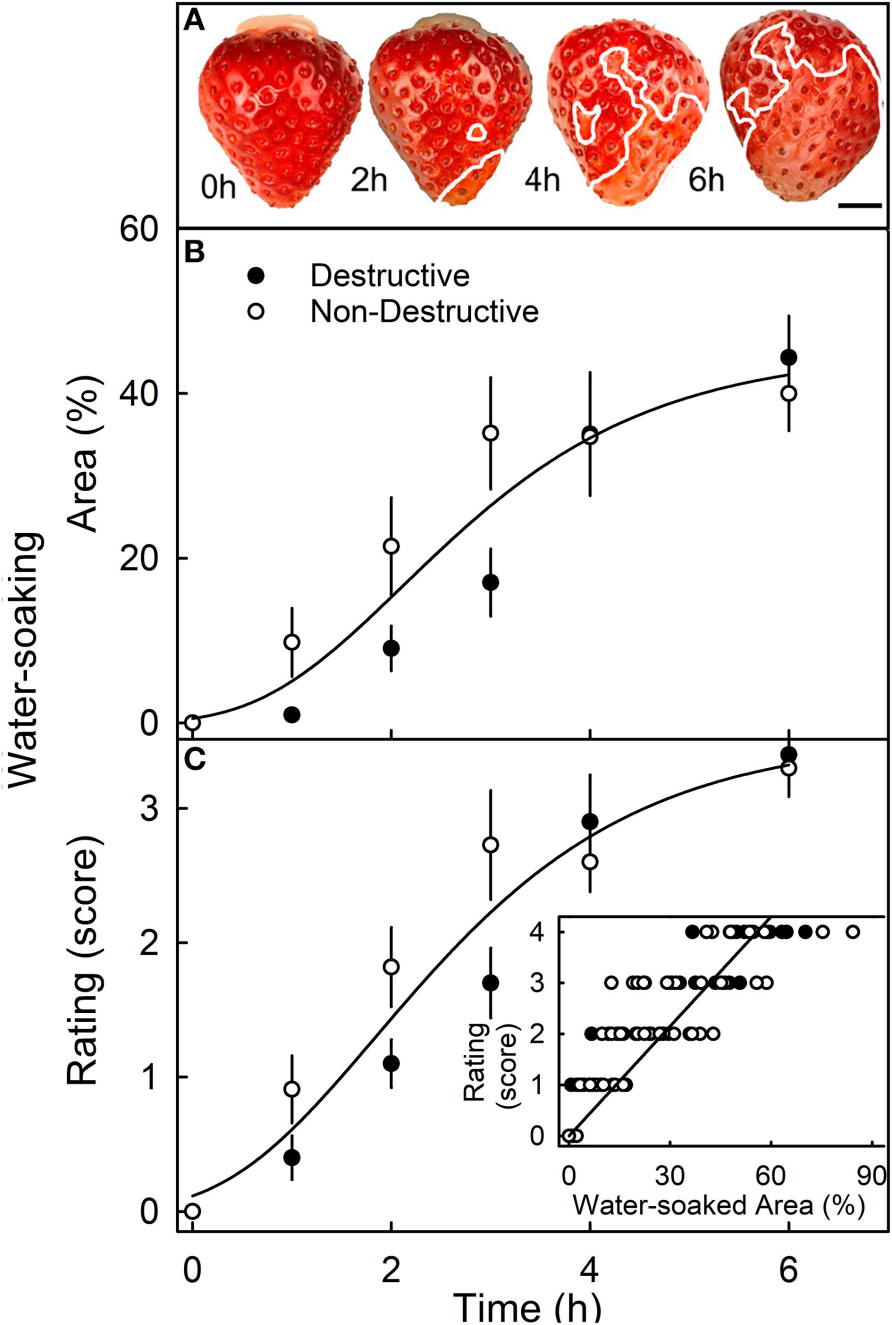
Time course of water soaking. **(A)** Macroscopic view of change in water-soaked area with time. The symptomatic area is indicated by a solid white line. Scale bar, 5 mm. **(B,C)** Change in water soaking as indexed by the measurement of the water-soaked area **(B)** or as assessed by a 5-point rating scale **(C)**. (**C**, inset): Relationship between the water-soaked score and the water-soaked area. For the destructive sampling depicted in **(B)**, an independent set of fruit was assessed at any one time. For the non-destructive sampling in **(B)**, repeated observations were made on the same fruit that was photographed repeatedly. The water-soaked area was quantified by digital analysis. The 5-point rating scale: score 0, no water soaking; score 1, <10% of the surface area water-soaked; score 2, 10–35%; score 3, 35–60%; score 4 > 60%.

When completely submerging fruit, the surface area affected by water soaking increased primarily because the water-soaked area expanded, and not because the number of water-soaked patches increased (Figure 3A). The surface area affected, increased sigmoidally with time until a major portion of the skin was affected (Figure 3B). There was no difference in the area affected or the rating score for water soaking between fruit that was sampled destructively and fruit that was monitored in a repeated-measures design (Figures 3B,C). Water soaking as indexed by the rating scheme was closely and significantly correlated with the water-soaked area as measured directly by image analysis (Figure 3C, inset).

**Figure 4:**
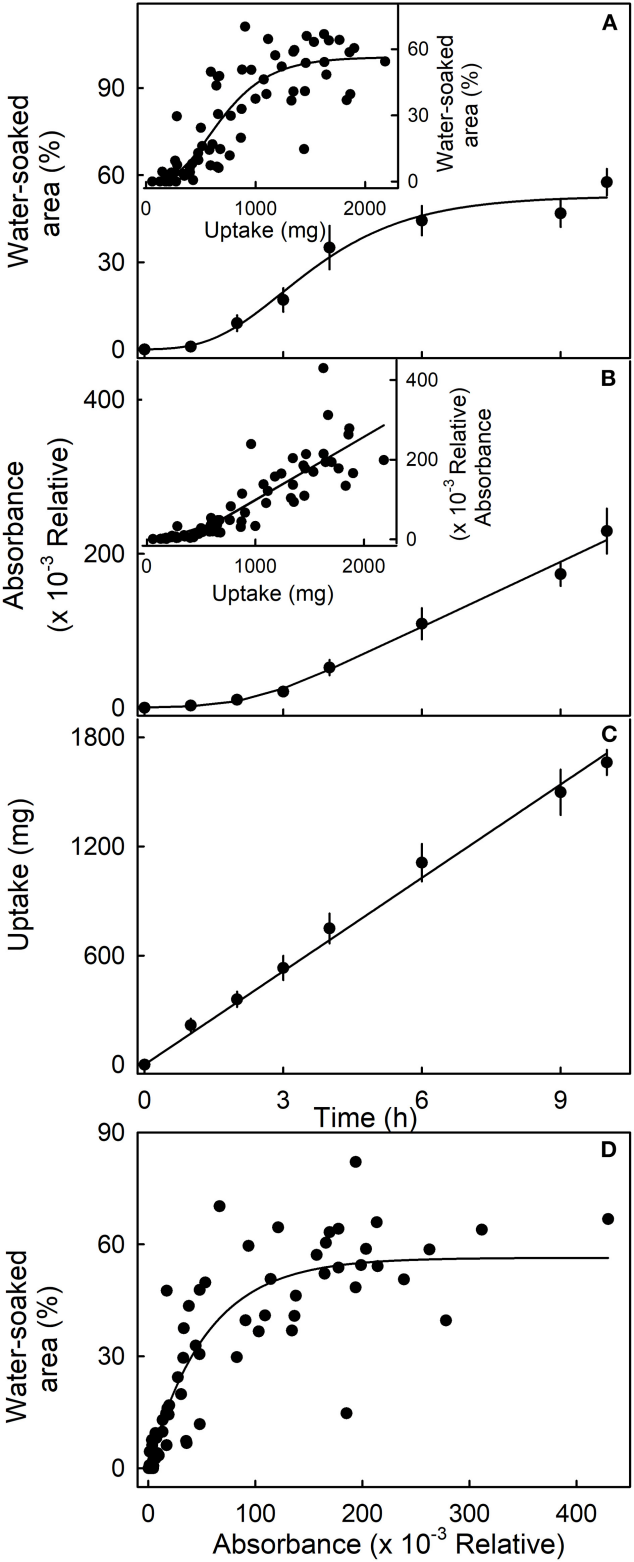
Time course of **(A)** change in water-soaked area, **(B)** change in anthocyanin leakage as indexed by absorbance of the incubation solution at 520 nm, and **(C)** water uptake of strawberries incubated in deionized water. Insets. Relationship between the water-soaked area (inset in **A**), the change in absorbance (inset in **B**), and water uptake. **(D)** Relationship between the water-soaked area and the leakage of anthocyanins as indexed by the change in absorbance.

The sigmoidal time course of the increase in water-soaked area had an initial lag phase where no symptoms appeared before water soaking began. Thereafter, the affected area increased (Figure 4). Similarly, following an initial lag phase, the leakage of anthocyanin increased. The leakage increased at a constant rate. However, for water uptake, there was no lag phase—water uptake began at the time of submersion and also accumulated at a constant rate (Figure 4C). When the water-soaked area or the leakage of anthocyanin (relative absorbance) was plotted vs. water uptake, a lag phase without water soaking and without anthocyanin leakage was present (Figures 4A,B, insets). These results indicate that water uptake increases up to some critical threshold beyond which water soaking and anthocyanin leakage begin. Water-soaked area and anthocyanin leakage were positively related until about 60% of the area was water-soaked. Above this value, anthocyanin leakage continued to increase, but water-soaked area remained largely constant (Figure 4D).

**Figure 5:**
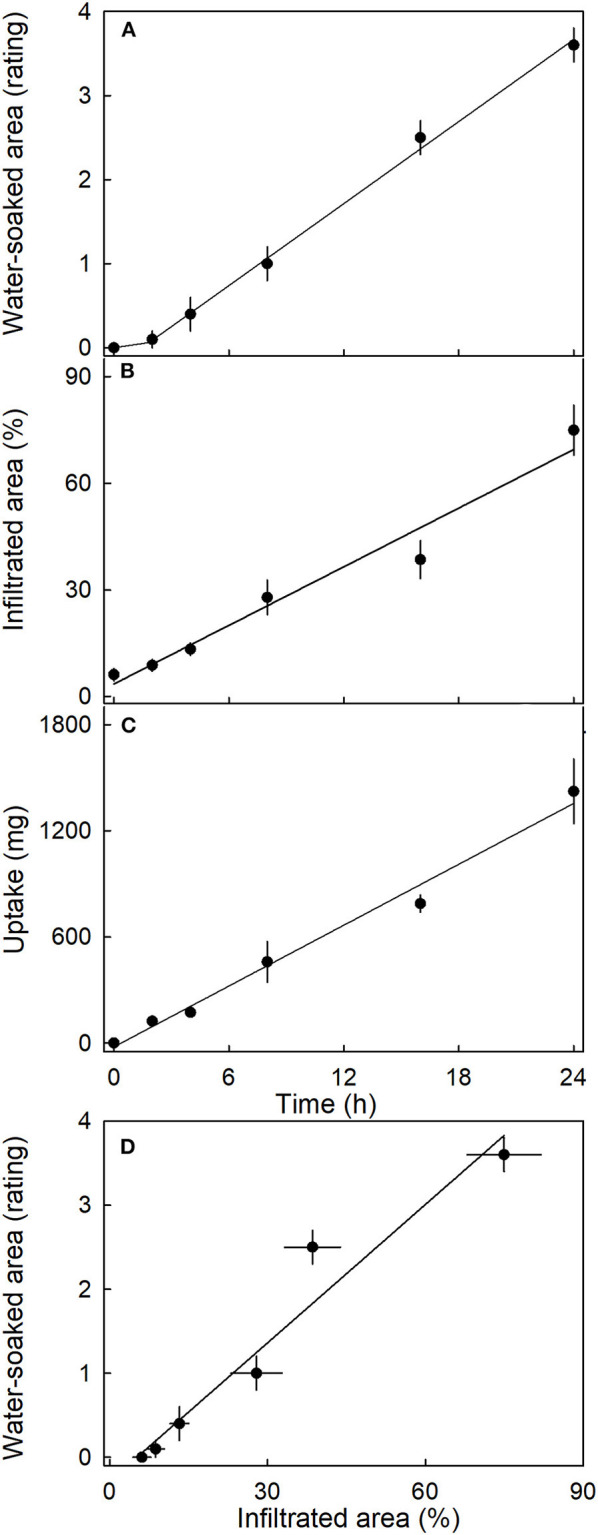
Time course of **(A)** change in water-soaked area, **(B)** change in the area infiltrated by acridine orange, and **(C)** water uptake of strawberries incubated in deionized water. **(D)** Relationship between the water-soaked area and the area infiltrated by acridine orange. Acridine orange penetrates the strawberry fruit skin *via* microcracks in the cuticle. Water soaking was indexed using a 5-point rating scale: score 0, no water soaking; score 1, <10% of the surface area water-soaked; score 2, 10–35%; score 3, 35–60%; score 4 > 60%.

The water-soaked area, the infiltrated area, and the water uptake all increased nearly linearly with time (Figure 5). The water-soaked area and the area infiltrated by the fluorescence tracer were significantly and positively related (*r*^2^= 0.95^***^; Figure 5D). It is worth noting that the rate of water uptake and the rate of water soaking were both markedly lower in “Clery” than in the cultivars used in other experiments.

**Figure 6:**
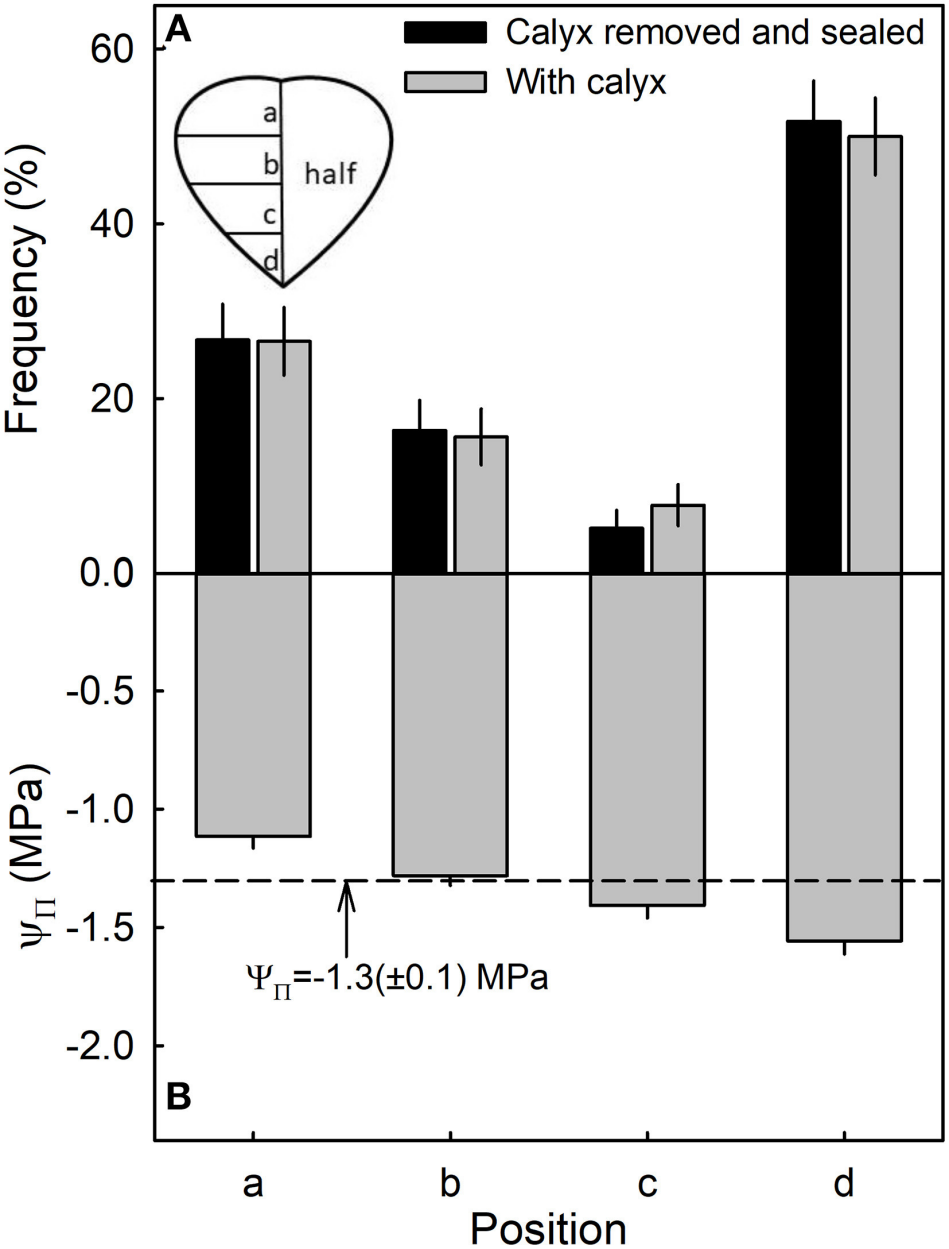
Frequency of symptoms of water soaking **(A)** and the osmotic potential (Ψ_Π_) of the juice **(B)** in different regions of a strawberry. The dashed line represents the mean osmotic potential of the fruit.

Most water soaking began at the tip of the fruit, followed by the calyx end, the calyx equator zone, and the equator tip zone (Figure 6A). There was no difference between fruit with or without the calyx. Within the fruit, osmotic potential decreased and became more negative from the calyx, toward the tip of the fruit (Figure 6B). There was no significant difference in stress relaxation between different regions of the fruit as indexed by a lack of difference in gaping following skin incision (data not shown). In all regions, the gap produced by the incision averaged about 0.95 ± 0.03 mm (*n* = 78) (Hurtado, unpublished data).

**Figure 7:**
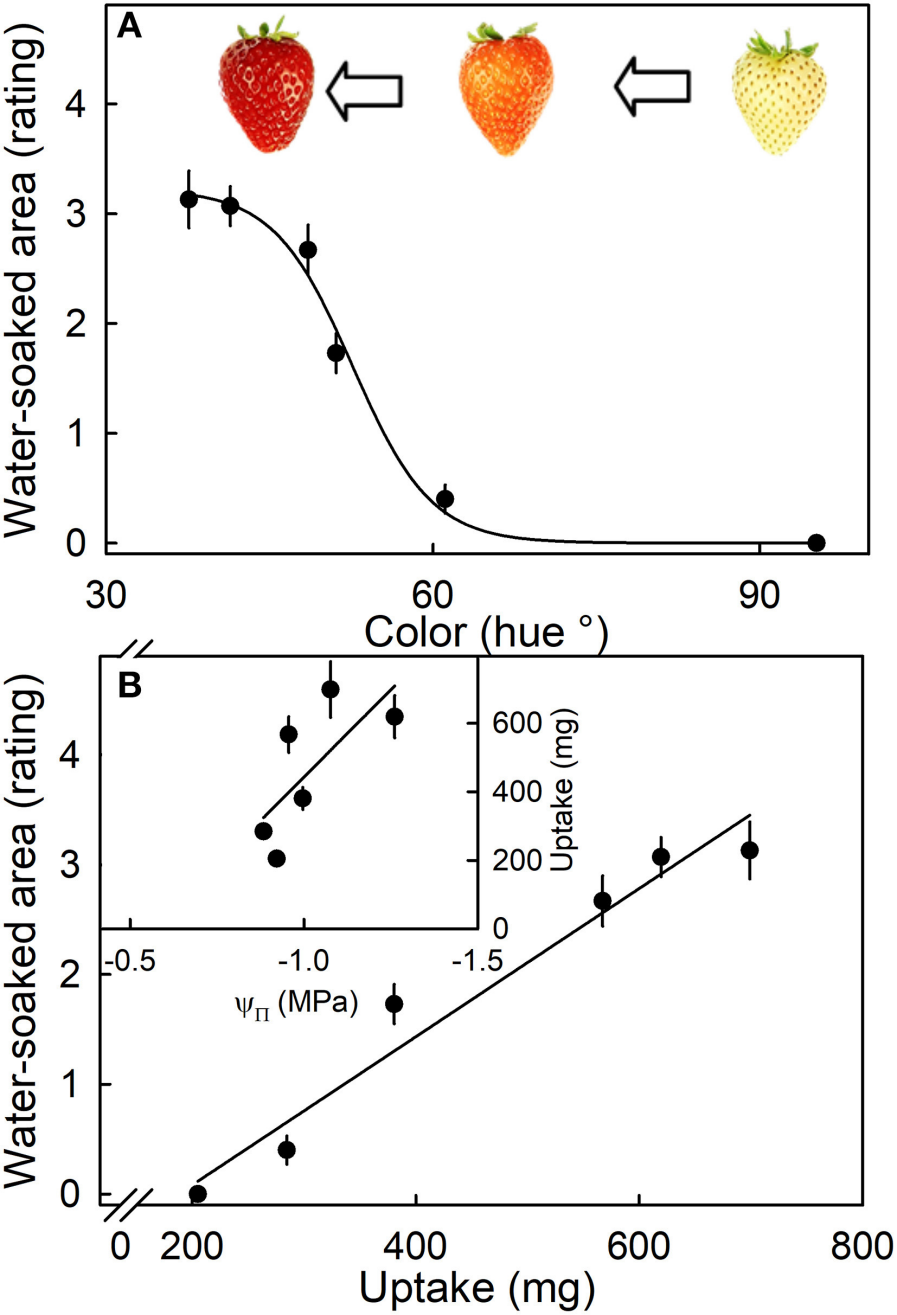
**(A)** Effect of ripening stage (as indexed by hue angle) on the water-soaked area when strawberries are incubated in deionized water. **(B)** Relationship between the water-soaked area and water uptake. Inset: Uptake vs. osmotic potential. Water soaking was indexed using a 5-point rating scale: score 0, no water soaking; score 1, <10% of the surface area water-soaked; score 2, 10–35%; score 3, 35–60%; score 4 > 60%.

The susceptibility to water soaking increased sigmoidally with ripening as indexed by the change in fruit color from white to dark red (Figure 7A). Across the different ripeness stages, the water-soaked area and water uptake rate were linearly related (Figure 7B, main graph). Unripe fruit (white) had the lowest values of both uptake and water soaking, while fully ripe fruit (dark red) showed the highest values. Accordingly, the relationship between osmotic potential and water uptake was linear and positive. The fully ripe, dark-red fruit also had the most negative osmotic potentials and the highest rates of water uptake (Figure 7B, inset).

**Figure 8:**
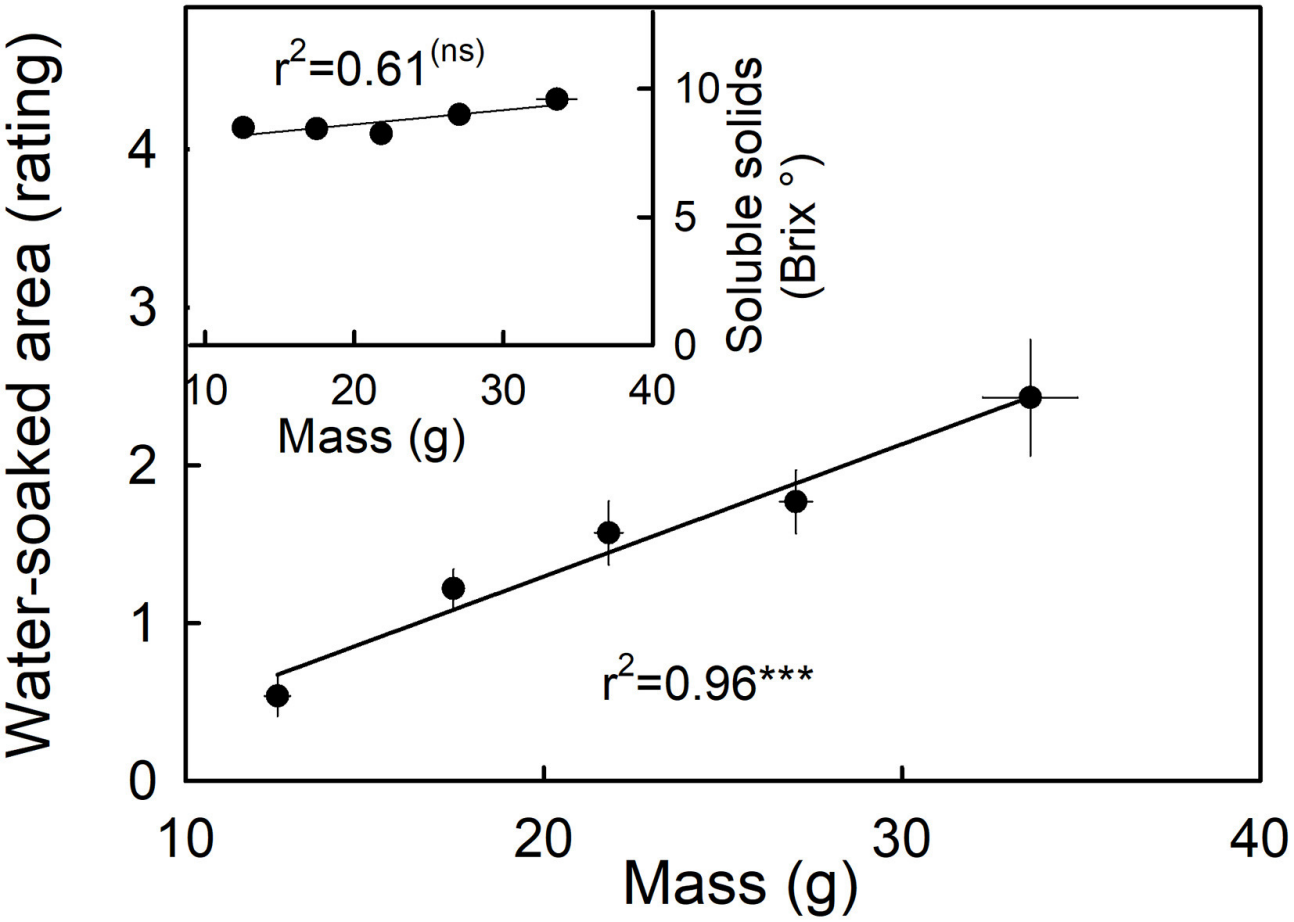
Relationship between water soaking and fruit mass. Inset: Relationship between the soluble solids as indicated by °Brix and water soaking. Water soaking was indexed using a 5-point rating scale: score 0, no water soaking; score 1, <10% of the surface area water-soaked; score 2, 10–35%; score 3, 35–60%; score 4 > 60%.

Water soaking increased as fruit mass increased (Figure 8, main graph). The largest fruit also had the highest rating scores and hence the largest portions of their surfaces water-soaked. Fruit mass was independent of the soluble solids content (Figure 8, inset).

**Figure 9:**
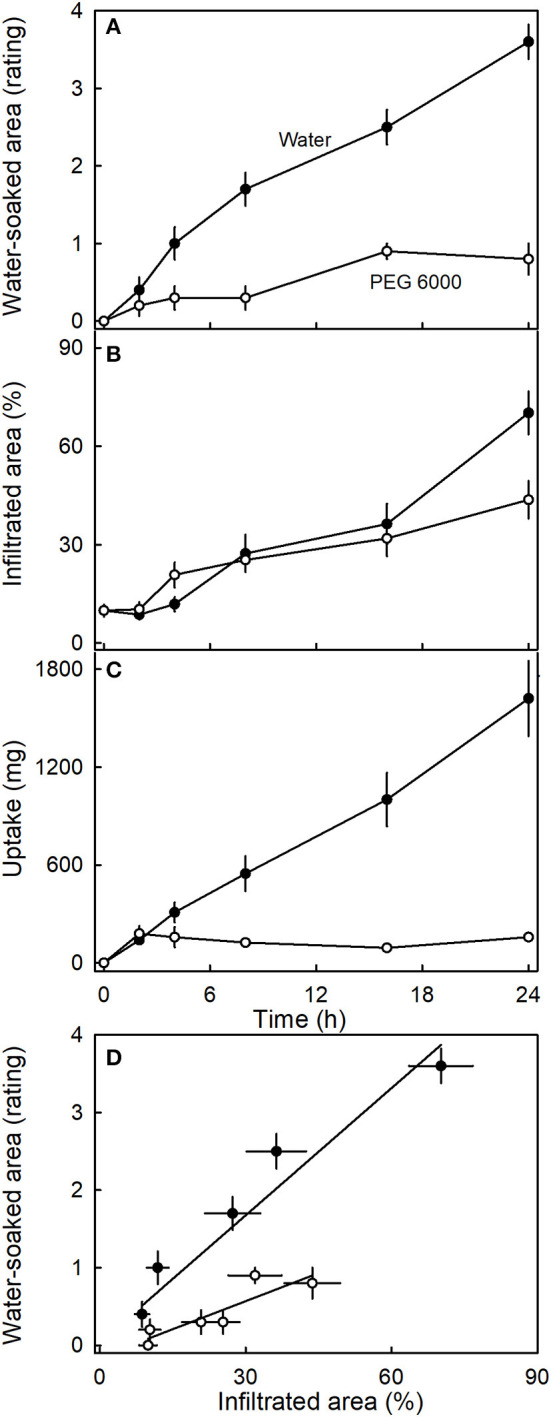
Time course of **(A)** change in water-soaked area, **(B)** change in the area infiltrated by acridine orange, and **(C)** water uptake of strawberries incubated in deionized water or in isotonic polyethylene glycol 6000 (PEG 6000). **(D)** Relationship between the water-soaked area and the area infiltrated by acridine orange. Acridine orange penetrates the strawberry fruit skin *via* microcracks in the cuticle. Water soaking was indexed using a 5-point rating scale: score 0, no water soaking; score 1, <10% of the surface area water-soaked; score 2, 10–35%; score 3, 35–60%; score 4 > 60%.

Incubating strawberries in isotonic PEG 6000 resulted in less water soaking and negligible water uptake compared to the control incubated in water (Figure 9). Interestingly, there was little difference in microcracking between fruit incubated in isotonic PEG 6000 or in water as indexed by the areas infiltrated by acridine orange (Figure 9B). The water-soaked area and the area infiltrated by acridine orange were positively related (Figure 9D).

Incubating strawberries in natural juice or artificial juice prepared at hypotonic, but not at isotonic concentrations, significantly increased water soaking. In contrast, water uptake decreased in both juices at hypotonic and even more so at isotonic concentrations as compared to the water control (Table 1). There were no differences either in water-soaked area or in water uptake between natural and artificial juice (data not shown). Interestingly, the rates of water uptake were consistently greater than zero, even though the incubation solution was isotonic to the fruit's expressed juice.

**Table 1:** Effect of natural (expressed) juice and artificial (compounded) juice on development of water soaking in “Faith” strawberry.

**Treatment**	**Water uptake (rate, mg h**^****–1****^**)** Osmotic potential (MPa)			**Water soaking (score, arbitrary) Osmotic potential (MPa)**		
	**0.5**	**1.0**	**Mean**	**0.5**	**1.0**	**Mean**
Artificial juice	104.3 ± 18.6	53.8 ± 12.4	78.9 ± 12.2^(ns)^	2.2 ± 0.2	1.6 ± 0.3	1.9 ± 0.2^(ns)^
Natural juice	83.2 ± 31.9	58.1 ± 13.0	70.7 ± 17.0	1.8 ± 0.2	1.5 ± 0.2	1.7 ± 0.1
Mean	93.8 ± 18.2a^a^	55.8 ± 8.8 b	74.8 ± 10.4	2.0 ± 0.1 a	1.5 ± 0 b	1.8 ± 0.1

*Water soaking was indexed using a 5-point rating scale: score 0, no water soaking; score 1, <10% of the surface area water-soaked; score 2, 10–35%; score 3, 35–60%; score 4 > 60%. In the deionized water control, the rate of water uptake was 122.8 ± 18.7 mg h^−1^, and the score for water soaking was 1.6 ± 0.2*.

*Analysis of variance revealed no significant interaction term but a significant main effect for osmotic potential*.

(ns) *Non-significant effect for treatment*.

a *Means followed by the same letter are not significantly different, Tukey's test at p = 0.05. ns, non-significant effect*.

Studying the effects of the individual components of strawberry juice on water soaking and water uptake revealed that citric and malic acids markedly increased water soaking compared to the water control. The rates of water uptake were higher compared to the other osmolytes or the water control. The carbohydrates that accounted for most of the osmolarity in strawberry juice decreased both water soaking and rates of water uptake to levels below the water control (Table 2).

**Table 2:** Effect of major osmolytes on the rate of water uptake and the development of water soaking in strawberries of cultivar “Florentina.”

**Component**	**Concentration (mM)**	**−Ψ_Π_ (Mpa)**	**pH**	**Rate of uptake (mg h^**−1**^)**	**Rating (score)**
Water	0.0	0.0	6.4	117.3 ± 13.8 b^a^	1.9 ± 0.2 b
Malic acid	25.2	0.1	2.6	207.6 ± 27.1 a	3.7 ± 0.1 a
Citric acid	43.4	0.1	2.3	176.4 ± 16.8 a	3.5 ± 0.2 a
Carbohydrates	316.6	0.8	6.5	63.0 ± 7.4 c	1.0 ± 0.2 c
Artificial juice	428	1.1	3.2	50.2 ± 5.2 c	1.6 ± 0.2 b

*Water soaking was indexed using a 5-point rating scale: score 0, no water soaking; score 1, <10% of the surface area water-soaked; score 2, 10–35%; score 3, 35–60%; score 4 > 60%. The artificial juice was compounded from the major osmolytes found in strawberries (together these account for 96.1% of the osmolarity of a strawberry) (Herrmann, 2001). The artificial juice was isotonic with the expressed natural juice*.

a *Means followed by the same letter are not significantly different, Tukey's test at p = 0.05*.

**Figure 10:**
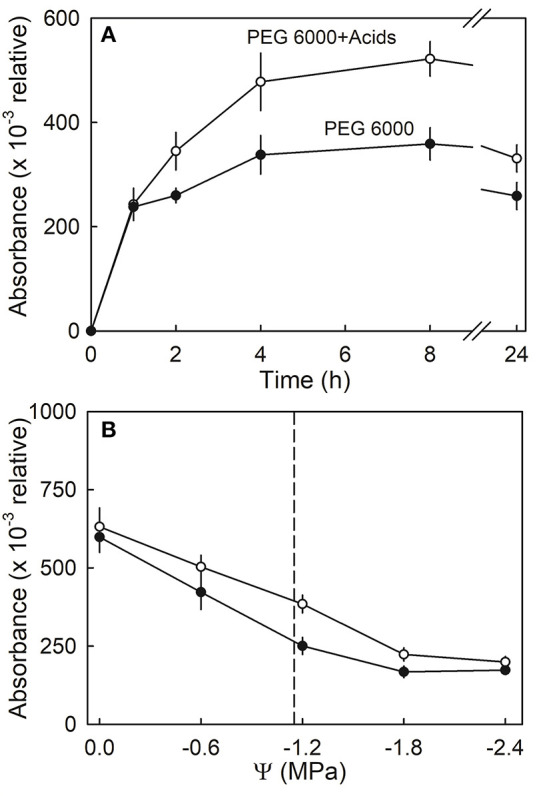
**(A)** Time course of anthocyanin leakage from flesh disks excised from ripe strawberries in the presence or absence of citric or malic acids. Flesh disks were incubated in isotonic polyethylene 6000 (PEG 6000) solutions. **(B)** Effect of osmotic potential (Ψ) of the incubation solution on anthocyanin leakage in the presence or absence of citric or malic acids. The vertical dashed line indicates the osmotic potential of juice expressed from the fruit of the same batch.

Anthocyanin leakage from flesh disks increased with time and was higher in the presence of citric and malic acid, than in the control (deionized water) (Figure 10A). The amounts of leakage were also higher from hypotonic than from hypertonic solutions. Again, leakage in the presence of the acids exceeded that in their absence. A two-factorial analysis of variance revealed the significant main effects for osmotic potential and the acids; there was no significant interaction term between these two factors (Figure 10B).

## Discussion

The main findings are as follows:

1. Water soaking involves microcracking of the cuticle, water uptake, bursting of cells, and leakage of cell contents
2. The behavior of water soaking in strawberry bears many similarities to the zipper model used to explain rain cracking in sweet cherries.

### Water Soaking Requires Microcracking of the Cuticle, Water Uptake, and the Bursting of Cells

This hypothesis is based on the following evidence.

(1) Surface wetness induced microcracking in the cuticle of the strawberry fruit when incubated in water in this and our earlier study (Hurtado et al., 2021) or in isotonic solutions (PEG 6000) (Figure 9). This is consistent with the literature reports of other fleshy fruit crops, including sweet cherry (Knoche and Peschel, 2006), apples (Knoche and Grimm, 2008; Khanal et al., 2021), *Ribes* berries (Khanal et al., 2011), and grapes (Becker and Knoche, 2012a). Microcracks impair the barrier properties of the cuticle. They represent a pathway for a rapid and localized water uptake by viscous flow through the strawberry fruit surface (Hurtado et al., 2021). Microcracking is an essential, but not the only requirement, in water soaking. For example, incubation in isotonic PEG 6000 induced microcracking, but markedly reduced water uptake and hence water soaking. Simulating microcracking by abrading the cuticle from the fruit surface induced water soaking like symptoms. After puncturing the fruit skin, the water-soaked area was much smaller. This is also consistent with the above hypothesis. It is important to note that microcracking of the cuticle due to moisture exposure also accounts for the localized nature of water soaking. Microcracks impair the cuticle's barrier function and allow a rapid, localized water uptake (Hurtado et al., 2021). This also explains why partial incubation of fruit resulted in water soaking only in the exposed regions.

(2) Water uptake markedly increased water soaking. Manipulations such as incubation in isotonic PEG 6000 that resulted in reduced water uptake also reduced water soaking. The only exceptions were treatments where the incubation solutions contained organic acids. The effect of organic acids is addressed in detail below. Also, more mature fruit, with more negative osmotic potentials and hence, higher rates of water uptake, also showed more water soaking. A more negative osmotic potential and hence, a higher rate of water uptake, is also consistent with a higher incidence of water soaking at the tip of the fruit. These results indicate that mature, ripe fruit is more susceptible to water soaking, particularly at the tip. The relatively high frequency of water soaking in the calyx end of the fruit (regions a, b) may have resulted from growth stress and strain that will be maximal in these regions. Additional evidence for a water uptake requirement comes from experiments where the fruit was incubated in artificial or natural juices. Here, water soaking was more severe in the hypotonic juices treatments, which had higher rates of water uptake, compared with the isotonic juices.

(3) Finally, bursting of cells is involved in water soaking. Evidence for this comes from the experiment on anthocyanin leakage. Here, up to some critical threshold, water uptake did not induce water soaking. However, beyond this threshold, water soaking began. The timing of the critical threshold for water uptake coincided with that for the onset of leakage. The latter indicates the onset of cell bursting. It is interesting to note that the release of organic acids that accompanies cell bursting seems to exacerbate water soaking. Experiments using natural or artificial juices and the major components thereof clearly identified citric and malic acids as the critical constituents that accelerate the development of water soaking. Both acids are released into the apoplast when a cell bursts. Here, they increase the permeability of the plasma membrane of neighboring cells. This causes cell leakage to propagate. Similar observations have been made for malic acid in sweet cherry, and these led to the development of the “Zipper model” where the acids “unzip” the skin (Winkler et al., 2015). In contrast to sweet cherry, in strawberry, citric and malic acids had no effect on the strength of the cell walls. This conclusion is inferred from the lack of a significant interaction between the osmotic potential of the incubation solution and the presence of the acids. At low (more negative) osmotic potentials, the incubation solutions were isotonic or hypertonic. A gradient for osmotic water uptake into the cells, and hence increased stress on the cell walls, must be absent. In contrast, at higher (less negative) osmotic potentials, the incubation solution was hypotonic, and hence, osmotic water uptake by the cells occurred. This imposes increased stress on the cell walls. However, the effect of the acids was independent of the applied stress on the cell walls. Hence, the acid effect must have been on the plasma membrane and not on the cell walls. This conclusion is also consistent with the wounding effect. Cuticle abrasion was more effective in inducing water soaking than either incisions or punctures. With abrasion, relatively large areas of the skin are damaged. It is the skin cells that contain most citric and malic acids (in the skin, the summed masses of both acids was 10.6 mg g^−1^) compared with about half this amount in the flesh (in the flesh, the summed masses of both acids were 5.5 mg g^−1^) (Holcroft and Kader, 1999).

The bursting of cells also offers a convincing explanation for the characteristic translucency of a water-soaked tissue. Water-soaked tissue typically results from the flooding of the gas-filled intercellular spaces (Sideris and Krauss, 1933). In addition, the leakage of both the tonoplast and the plasma membranes results in a general mixing of apoplastic and symplastic components (the anthocyanins are naturally contained within the tonoplast—vacuole). The apoplast pH is likely to increase, compared to that of the vacuole. As a result, the anthocyanins will likely react with water, to form colorless pseudobases (Brouillard et al., 1997; Holcroft and Kader, 1999). This explains the lighter color of a water-soaked tissue.

### The Mechanism of Water Soaking Is Similar to That of Cracking in Sweet Cherry Fruit

Water soaking in strawberry can be substantially explained in terms of the zipper model that accounts for sweet cherry fruit cracking during/following rainfall (Winkler et al., 2016). Based on this model, a series of sequential events leads to water soaking. First, the barrier function of the cuticle is impaired by the formation of microcracks in response to surface wetness (Figure 2). Because the cuticle of a strawberry is very thin (Hurtado et al., 2021), microcracks allow a rapid, localized water uptake into the subtending tissues. This uptake occurs by viscous flow and is more rapid than that by diffusion through an intact cuticle (Hurtado et al., 2021). Unlike in apple, there is no evidence of any repair processes in strawberry: wax deposition in a microcrack (Curry, 2009) or the formation of a periderm (Evert, 2006; Macnee et al., 2020). Second, water uptake causes cells to burst, as indicated by the leakage of anthocyanin. Because strawberries contain large amounts of citric and malic acid, particularly in the skin cells, and because these acids increase the permeability of plasma membranes, the neighboring cells also begin to leak. This propagation process causes the water-soaked area to spread both laterally and down into the fruit.

Unlike in sweet cherry, there is no evidence in strawberry that these organic acids cause a weakening of cell walls. Nevertheless, future evidence for a weakening would not be surprising, as both citric and malic acids are likely to extract Ca from the cell walls, and this loss will decrease cell-to-cell adhesion (Winkler et al., 2015). However, at this stage, there is no support for such effects in strawberry.

## Conclusions

Our results demonstrate that water soaking in strawberry results from a series of events that involves microcracking of the cuticle, water uptake, a cell bursting, and the release of organic acids into the apoplast. In many ways, it is similar to rain cracking in fleshy fruit, such as sweet cherry. Based on our findings, a reliable strategy to prevent water soaking would be to limit a direct contact between water and the fruit surface. This may be achieved preharvest by protected cultivation, either in a greenhouse or in a plastic tunnel. Important measures postharvest include the use of appropriate packaging materials and handling practices that avoid the occurrence of liquid water on the surface, for example, due to condensation of water vapor. In the long run, the susceptibility of commercial strawberry genotypes to water soaking may be decreased by breeding. Our results and those by Herrington et al. (2009) demonstrate that susceptibility to water soaking differs among cultivars. Water soaking occurred in all cultivars used in our study, but at a markedly lower rate in “Clery” as compared to “Faith” and “Florentina.” The basis of these differences has not yet been identified. Among the three cultivars, “Clery” also had the lowest rate of water uptake, which probably contributed to the lower rate of water soaking. Based on the findings presented herein and their similarity to the phenomenon of rain cracking in sweet cherry, potentially useful aims for further research in water soaking are to determine (a) the pattern of cuticle deposition in developing strawberry fruit, (b) the water uptake characteristics, (c) the mechanical strength of their cell walls, and (d) the strength of their cell-to-cell adhesion. A better understanding of the phenomenon of microcracking would also be beneficial in reducing the incidence of fruit rots. Pathogens like *Botrytis cinerea* benefit from microcracks in the cuticle because of the impaired barrier functions (Jarvis, 1962).

## Data Availability Statement

The raw data supporting the conclusions of this article will be made available by the authors, without undue reservation.

## Author Contributions

MK obtained the funds to support the study. GH and MK planned the experiments, analyzed the data, wrote, revised, and edited the manuscript. GH conducted the experiments. All authors contributed to the article and approved the submitted version.

## Conflict of Interest

The authors declare that the research was conducted in the absence of any commercial or financial relationships that could be construed as a potential conflict of interest.
