# Supplementary material for: Water Soaking Disorder in Strawberries: Triggers, Factors, and Mechanisms
Source: Front Plant Sci. 2021 Jul 20;12:694123. doi: 10.3389/fpls.2021.694123 (PMC8330803; doi:10.3389/fpls.2021.694123)
Supplement: Supplementary file 1 [file Presentation_1.PDF]

Supplementary Material

1.1 Supplementary Image

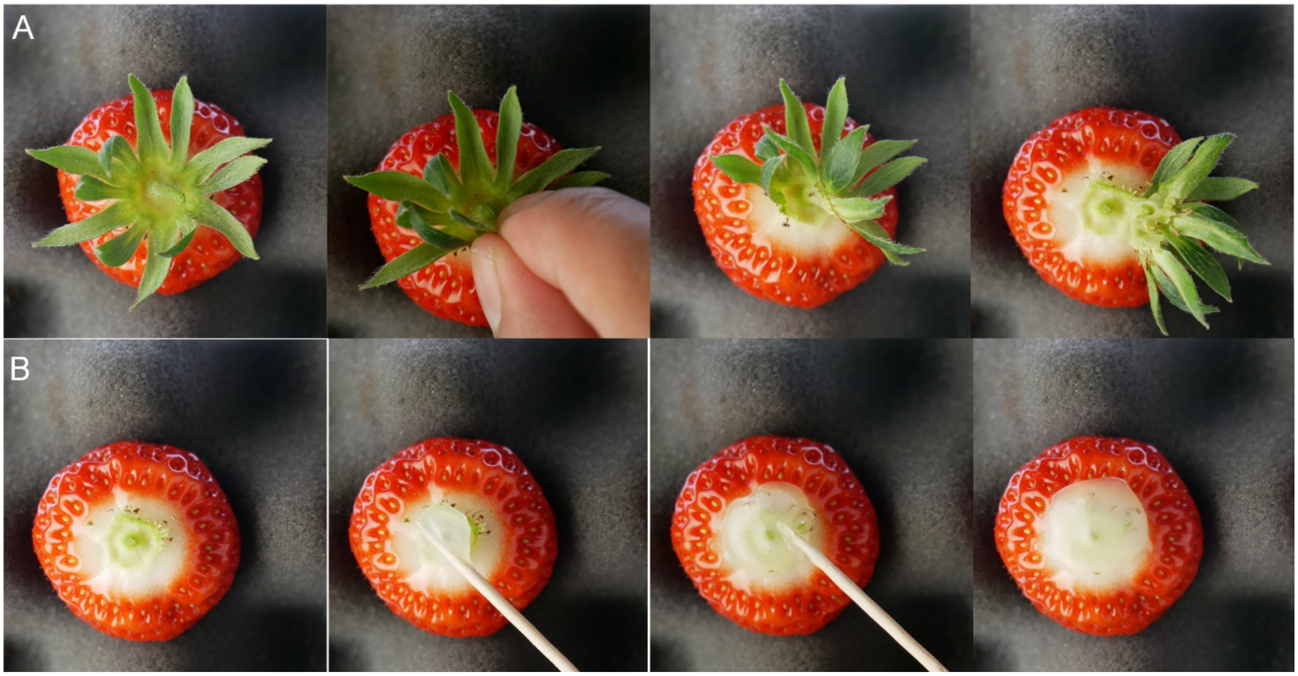

Supplementary Figure S1. Procedure of calyx extractions and sealing. **(A)** Manual extraction of calyx. **(B)** Sealing of the remaining calyx base and pedicel stub with silicone rubber.
